# Thrips (Insecta, Thysanoptera) of Iran: a revised and updated checklist

**DOI:** 10.3897/zookeys.330.5939

**Published:** 2013-09-11

**Authors:** Kambiz Minaei

**Affiliations:** 1 Department of Plant Protection, College of Agriculture, Shiraz University, Shiraz, Iran

**Keywords:** Iran, list, species, Thysanoptera

## Abstract

In Iran, as a result of recent changes in nomenclature 201 species and one species group of the insect Order Thysanoptera, are here listed in 70 genera and five families. In considering species listed previously from this country, the presence of 7 species is considered not confirmed, and 12 species are excluded from the Iranian list. Problems in the study of Iranian Thysanoptera are discussed briefly.

## Introduction

Iran forms a large part of the Iranian plateau, and covers an area of 1,623,779 km². It is bordered in the north by the Caucasus Mountains, Middle Asian natural regions and the Caspian Sea (-27 m below sea level); in the west by the Anatolian and Mesopotamian regions; in the east by the eastern part of the Iranian plateau (Afghanistan and adjacent west Pakistan) and the Baluch-Sindian region; and finally in the south by the Persian Gulf and Oman Sea, which are connected by the latter to the Indian Ocean (Zehzad et al. 2002).

In Iran, the first record of thrips species was of three species, *Frankliniella intonsa* (Trybom), *Thrips flavus* Schrank and *Thrips tabaci* Lindeman, as pests of summer crops (Afshar 1938), and after that there were several scattered studies of this group in various parts of this country. Recently the extensive Iranian literature on these insects was summarised by Bhatti et al. (2009a), who listed 177 species in 62 genera. However that checklist needs further consideration for four reasons:

1. The checklist by Bhatti et al. (2009a) covers the literature until 2007 and since then several important works on Thysanoptera of Iran have been published, including a further 13 genera and 38 species recorded or described. Moreover, a few recent name changes have become available.
2. There are some misinterpretations of “Iranian Persian literature” in Bhatti et al. (2009a). Thus a few species have appeared in Iranian literature as potential pests or as exotic pests without any supporting data or records from Iran.
3. Bhatti et al. (2009a) did not employ the standard suprageneric classification of Thysanoptera, so the utility of the checklist for students is limited.
4. The restricted distribution of the journal in which the book (Bhatti et al. 2009a) was published limits its utility to entomologists in Iran as well as the world.

## Thrips studies in Iran: problems

Relevant information about thrips species recorded from Iran is severely lacking. For example, until the end of 2007, 187 primary references had been published on Iranian Thysanoptera, but, of these, 123 (65%) appeared only as “abstracts”. Almost all of these consisted solely of species lists, without any further information being provided as to the number of collected specimens, their sex, or the habitats in which the species were collected. In one of these abstracts (Mortazaviha 1995) even the specific locality where the species were collected is not given, and for 15 thrips species collection details are restricted to the country “Iran”. A further problem is the difficulty in tracing collections in which relevant voucher specimens were placed; and for many species there appear to be no extant voucher specimens. For example, *Haplothrips minutus* was recorded by Kheyrandish Koshkoei et al. (2000), but when asked for a loan of material Kheyrandish Koshkoei (personal communication 2006) responded that he did not have access to any specimens of that species. Similarly, two papers (Mehrnejad and Panahi 2006; Kazemi and Mehrnejad 2011) concerning the biology and pest status of *Liothrips austriacus* (Karny) have been published from work carried out at the “Pistachio Research Institute” in Rafsenjan, Kerman Province, but no specimens of that species are available from that Institute at present (F. Kazemi, personal communication, 2013). Furthermore, in recent years Majid Mirab-balou has described or recorded several thrips species from Iran, but the specimens (including type specimens) have been deposited in China (Mirab-balou and Chen 2012a, b).

The third problem, related to the above, is imprecise reporting by Iranian authors. Several species have been reported from Iran despite the original specific identifications on which these reports are based remaining tentative. For example, the Iranian records for three *Haplothrips* species reported by Bagheri and Alavi (2007) are based on specimens identified by zur Strassen as “perhaps” those species (Minaei and Mound 2008). Similarly, three *Aeolothrips* species recorded by Fallahzadeh et al. (2011) were only tentatively identified to species by Bhatti (Minaei 2013a).

## A revised checklist of Thysanoptera from Iran

The following checklist is organized following the standard taxonomic hierarchy, and is based on published literatures including Bhatti et al. (2009a). For each suprageneric category a brief description is provided based largely on the Iranian fauna. Higher level taxonomy in the checklist follows Mound (2011a). Nomenclature follows that used in a web-based world checklist (ThripsWiki 2013), which should also be referred to for full synonymies for the names listed here. The checklist includes references for all additions and changes in taxonomic status or changes in synonymy made since the publication of the previous checklist by Bhatti et al. (2009a), and the symbol + is used to indicate these changes.

### Suborder Terebrantia

The Terebrantia comprises eight families (Mound 2011a) of which four (Aeolothripidae, Melanthripidae, Stenurothripidae, Thripidae) are represented in Iran. Terebrantia species are largely phytophagous, feeding in flowers and on leaves.

### Family Aeolothripidae

The family includes 194 extant species in 23 genera (ThripsWiki 2013), mostly from the temperate areas of the northern and southern hemispheres. Adults and larvae of many species in this family appear to be facultative predators of other small arthropods, in that they feed on both floral tissues as well as on thrips and mites that live in flowers. However, some species are almost certainly solely phytophagous, a few being univoltine in flowers of particular plant species (Tyagi et al. 2008), whereas in the warmer parts of the world, a considerable number of species are obligate predators (Hoddle 2003).

In this family, the most species-rich genus, *Aeolothrips* was interpreted by Bhatti (1988) in a different way to other specialists, with *Aeolothrips* restricted to *Aeolothrips albicinctus* Haliday. Bhatti’s interpretation put all other species from the original genus into four further genera (*Arabthrips* Bhatti, *Coleothrips* Haliday, *Fabothrips* Bhatti, *Podaeolella* Priesner). In this paper, that interpretation is not accepted. Four genera including 23 species are recognized in this family in Iran.

***Aeolothrips* Haliday, 1836**

+ *Aeolothrips afghanus* Jenser, 1984 male described by Minaei et al. (2013)

*Aeolothrips albicinctus* Haliday, 1836

*Aeolothrips collaris* Priesner, 1919

+ *Aeolothrips cursor* Priesner, 1939 added by Mirab-balou and Chen (2012c)

*Aeolothrips deserticola* Priesner, 1929

+ *Aeolothrips eremicola* Priesner, 1938 added by Zolfaghari et al. (2012); male de scribed by Alavi et al. (2013)

*Aeolothrips fasciatus* (Linnaeus, 1758)

*Aeolothrips gloriosus* Bagnall, 1914

*Aeolothrips heinzi* zur Strassen, 1990

*Aeolothrips intermedius* Bagnall, 1934

+ *Aeolothrips modestus* zur Strassen, 1966 added by Minaei (2013a)

*Aeolothrips mongolicus* Pelikan, 1985

+ *Aeolothrips montivagus* Priesner, 1948 added by Mirab-balou and Chen (2012c)

*Aeolothrips tenuicornis* Bagnall, 1926

*Aeolothrips versicolor* Uzel, 1895

+ *Aeolothrips wittmeri* Priesner, 1935 added by Alavi et al. (2012)

+ *Aeolothrips zurstrasseni* Minaei described by Minaei (2013a)

***Indothrips* Bhatti, 1967**

*Indothrips bhushani* Bhatti, 1967

***Orothrips* Moulton, 1907**

*Orothrips priesneri* (Titschack, 1958)

***Rhipidothrips* Uzel, 1895**

*Rhipidothrips brunneus* Williams, 1913

*Rhipidothrips flavus* Tunç, 1991

*Rhipidothrips gratiosus* Uzel, 1895

*Rhipidothrips unicolor* zur Strassen, 1965

### Family Melanthripidae

Melanthripids were considered to be members of the Aeolothripidae until recently. The family now includes 66 extant species in four genera: *Ankothrips* (13 species), *Cranothrips* (11 species), *Dorythrips* (6 species) and *Melanthrips* (35 species). All species in the family are flower-feeding but each genus exhibits a remarkable discontinuity in geographical distribution: *Cranothrips* and *Dorythrips* are known only from the Southern Hemisphere, whereas *Ankothrips* and *Melanthrips* are mainly from the Northern Hemisphere but each with one or two species from South Africa (Pereyra and Mound 2009, Hoddle et al. 2013). In Iran, seven species in two genera have been recorded.

***Ankothrips* Crawford, 1909**

+ *Ankothrips zayandicus* Minaei, Haftbaradarn & Mound, 2012 described by Minaei et al. (2012)

***Melanthrips* Haliday, 1836**

*Melanthrips fuscus* (Sulzer, 1776)

+ *Melanthrips hei* Mirab-balou & Chen, 2012 described by Mirab-balou and Chen (2012a)

*Melanthrips knechteli* Priesner, 1936

*Melanthrips pallidior* Priesner, 1919

*Melanthrips rivnayi* Priesner, 1936

*Melanthrips separandus* Priesner, 1936

### Family Stenurothripidae

The extant species in this group were placed in the family Adiheterothripidae (Bhatti 1986), but this is now considered a synonym of Stenurothripidae (Bhatti 2006). The three extant genera of this family occur in California and in the Mediterranean region through to India (Mound and Marullo 1999). The species in this family apparently all breed in flowers, and they probably have a high degree of host specificity. All four species of *Holarthrothrips* breed in the male flowers of date palms and its relatives (Mound et al. 2013b). Only one species is recorded in Iran.

***Holarthrothrips* Bagnall, 1927**

*Holarthrothrips josephi* Bhatti, 1986

### Family Thripidae

Thripids include 2020 species in 284 genera worldwide (ThripsWiki 2013). Most of them are phytophagous on higher plants, with a few species on ferns (Mound 2002), and a few are obligate predators (Mound 2011b). However, some polyphagous pest thrips such as *Frankliniella occidentalis* and *Thrips tabaci* can behave as facultative predators (Wilson et al. 1996). One genus in Brazil comprises species that are ectoparasitic on Hemiptera (Cavalleri et al. 2010). Four subfamilies within the Thripidae are currently recognized worldwide, and each of these is represented in Iran.

### Thripidae—Dendrothripinae

More than 90 species, in 11 genera, are recognized worldwide in this subfamily (ThripsWiki 2013). All of the species live on leaves. Five species in two genera have been recorded in Iran.

***Dendrothrips* Uzel, 1895**

*Dendrothrips degeeri* Uzel, 1895

*Dendrothrips karnyi* Priesner, 1921

*Dendrothrips phyllireae* (Bagnall, 1927)

*Dendrothrips saltator* Uzel, 1895

***Pseudodendrothrips* Schmutz, 1913**

*Pseudodendrothrips mori* (Niwa, 1908)

### Thripidae—Panchaetothripinae

Wilson (1975) provided an account of the members of this subfamily that is now considered to include 136 species in 38 genera. The species in this subfamily are all leaf feeding usually associated with older, senescing leaves (Mound et al. 2013b). Seven species in six genera have been found in Iran so far.

***Caliothrips* Daniel, 1904**

*Caliothrips impurus* (Priesner, 1928)

+ *Caliothrips quadrifasciatus* the species is recorded as *Caliothrips graminicola* Bagnall & Cameron, 1932 in Iranian literature

***Heliothrips* Haliday, 1836**

*Heliothrips haemorrhoidalis* (Bouche, 1833)

***Parthenothrips* Uzel, 1895**

*Parthenothrips dracaenae* (Heeger, 1854)

***Retithrips* Marchal, 1910**

*Retithrips syriacus* (Mayet, 1890)

***Rhipiphorothrips* Morgan, 1913**

*Rhipiphorothrips cruentatus* Hood, 1919

***Selenothrips* Karny, 1911**

+ *Selenothrips rubrocinctus* (Giard, 1901) added by Mirab-balou and Chen (2012d)

### Thripidae—Sericothripinae

This group is treated as a subfamily of Thripidae to include three genera: *Hydatothrips* Karny, *Neohydatothrips* John, *Sericothrips* Haliday (ThripsWiki 2013). This subfamily includes 148 species worldwide, and these are usually found in association with flowers but with some species breeding on leaves (Mound and Tree 2009). Bhatti (2006) proposed *Papiliothrips* as a new genus and transferred *Neohydatothrips gracilicornis* and two species of *Sericothrips* to the genus, but this is not accepted here. Two species in one genus are reported in Iran so far.

***Neohydatothrips* John, 1929**

*Neohydatothrips gracilicornis* (Williams, 1916)

*Neohydatothrips tadzhicus* (Pelikan, 1964)

### Thripidae—Thripinae

This is the largest group of Thripidae with 1644 species in 232 genera (ThripsWiki 2013). The species exhibit a wide range of biologies, and most of the species of thrips regarded as pests are included in this subfamily (Mound 1997). Bhatti et al. (2009a) synonymized *Chirothrips aculeatus* Bagnall with *Chirothrips pedestris* (Karny). However, this was not accepted by other researchers (Minaei and Mound 2010a). Moreover, in contrast to zur Strassen (2003) and Bhatti et al. (2009a), three *Chirothrips* species recorded in Iran (*Chirothrips africanus* Priesner, *Chirothrips manicatus* (Haliday), *Chirothrips pallidicornis* Priesner), together with *Chirothrips ammophilae* Bagnall, were placed in a *Chirothrips manicatus* species group by Minaei and Mound (2010a) due to the difficulty in separating them from each other by morphological characters, and this approach is accepted here. Two species listed under the genus *Thrips*, *Thrips iranicus* Yakhontov and *Thrips pistaciae* Yakhontov, are not recognizable at present due to the poor descriptions (see Bhatti et al. 2009a). In addition, *Thrips fraudulentus* (Priesner) is very similar to *Thrips atratus* Haliday. Laurence Mound (personal communication 2010) examined the holotype of *Thrips fraudulentus* in the Forschungsinstitut Senckenberg, Frankfurt, and although there are differences in the lengths of the antennae the recognition of *Thrips fraudulentus* as a distinct species remains doubtful. In Iran, 110 species and one species-group in 35 genera in this subfamily are recognized.

***Agalmothrips* Priesner, 1965**

*Agalmothrips parviceps* (Priesner, 1965)

***Anaphothrips* Uzel, 1895**

+ *Anaphothrips obscurus* (Müller, 1776) male described by Mirab-balou and Chen (2010)

*Anaphothrips sudanensis* Trybom, 1911

***Aptinothrips* Haliday, 1836**

*Aptinothrips elegans* Priesner, 1924

*Aptinothrips rufus* (Haliday, 1836)

*Aptinothrips stylifer* Trybom, 1894

***Arorathrips* Bhatti, 1990**

+ *Arorathrips mexicanus* (Crawford DL, 1909) added by Minaei and Alichi (in press)

***Bregmatothrips*** Hood, 1912

*Bregmatothrips bournieri* Pelikan, 1988

***Chirothrips* Haliday, 1836**

*Chirothrips aculeatus* (Bagnall, 1927)

+ *Chirothrips atricorpus* (Girault, 1927) stat. rev. by Minaei and Mound (2010a)

*Chirothrips kurdistanus* zur Strassen, 1967

+ *Chirothrips manicatus* species-group defined by Minaei and Mound (2010a)

+ *Chirothrips maximi* Ananthakrishnan, 1957 added by Mirab-balou et al. (2013)

+ *Chirothrips meridionalis* Bagnall, 1927 stat. rev. by Minaei and Mound (2010a)

*Chirothrips molestus* Priesner, 1926

***Collembolothrips* Priesner, 1935**

*Collembolothrips mediterraneus* Priesner, 1935

***Drepanothrips* Uzel, 1895**

*Drepanothrips reuteri* Uzel, 1895

***Eremiothrips* Priesner, 1950**

*Eremiothrips antilope* (Priesner, 1923)

*Eremiothrips arya* (zur Strassen, 1975)

+ *Eremiothrips bhattii* Minaei, 2012 described by Minaei (2012)

*Eremiothrips dubius* (Priesner, 1933)

*Eremiothrips efflatouni* (Priesner, 1965)

*Eremiothrips farsi* Bhatti & Telmadarraiy, 2003

*Eremiothrips shirabudinensis* (Yakhontov, 1929)

+ *Eremiothrips similis* Bhatti, 1988 added by Ramezani et al. (2009)

*Eremiothrips taghizadehi* (zur Strassen, 1975)

*Eremiothrips tamaricis* (zur Strassen, 1975)

*Eremiothrips varius* (Bhatti, 1967)

+ *Eremiothrips zurstrasseni* Bhatti, Bagheri & Ramezani, 2009 described by Bhatti et al. (2009b)

***Euphysothrips* Bagnall, 1926**

*Euphysothrips minozzii* Bagnall, 1926

***Exothrips* Priesner, 1939**

*Exothrips redox* Bhatti, 1975

***Ficothrips* Minaei, 2012**

+ *Ficothrips moundi* Minaei, 2012 described by Minaei (2012a)

***Frankliniella* Karny, 1910**

*Frankliniella intonsa* (Trybom, 1895)

*Frankliniella occidentalis* (Pergande, 1895)

*Frankliniella pallida* (Uzel, 1895)

*Frankliniella schultzei* (Trybom, 1910)

*Frankliniella tenuicornis* (Uzel, 1895)

***Kakothrips* Williams, 1914**

+ *Kakothrips dentatus* Knechtel, 1939 added by Mirab-balou and Chen (2011a)

*Kakothrips pisivorus* (Westwood, 1880)

*Kakothrips priesneri* Pelikan, 1965

***Limothrips* Haliday, 1836**

*Limothrips angulicornis* Jablonowski, 1894

+ *Limothrips cerealium* (Haliday, 1836) added by Mirab-balou et al. (2013)

*Limothrips denticornis* (Haliday, 1836)

*Limothrips schmutzi* Priesner, 1919

*Limothrips transcaucasicus* Savenko, 1944

***Megalurothrips* Bagnall, 1915**

+ *Megalurothrips distalis* (Karny, 1913) added by Mirab-balou and Chen (2011b)

***Microcephalothrips* Bagnall, 1926**

*Microcephalothrips abdominalis* (Crawford DL, 1910)

***Mycterothrips* Trybom, 1910**

*Mycterothrips consociatus* (Targioni-Tozzetti, 1887)

+ *Mycterothrips hamedaniensis* Mirab-balou, Shi & Chen, 2011 described by Mirab-balou et al. (2011)

*Mycterothrips latus* (Bagnall, 1912)

*Mycterothrips salicis* (Reuter, 1879)

*Mycterothrips tschirkunae* (Yakhontov, 1961)

+ *Mycterothrips weii* Mirab-balou, Shi & Chen, 2011 described by Mirab-balou et al. (2011)

***Odontothrips* Amyot & Serville, 1843**

*Odontothrips confusus* Priesner, 1926

*Odontothrips loti* (Haliday, 1852) added by Mirab-balou and Chen (2011b)

*Odontothrips meliloti* Priesner, 1951

*Odontothrips phlomidinus* Priesner, 1954

***Oxythrips* Uzel, 1895**

+ *Oxythrips claripennis* Priesner, 1940 added by Mirab-balou and Chen (2013)

*Oxythrips halidayi* Bagnall, 1924

*Oxythrips retamae* (Priesner, 1934)

*Oxythrips ulmifoliorum* (Haliday, 1836)

*Oxythrips wiltshirei* Priesner, 1954

***Parascolothrips* Mound, 1967**

*Parascolothrips priesneri* Mound, 1967

***Pezothrips* Karny, 1907**

*Pezothrips bactrianus* (Pelikan, 1968)

***Psilothrips* Hood, 1927**

*Psilothrips bimaculatus* (Priesner, 1932)

***Rubiothrips* Schliephake, 1975**

+ *Rubiothrips parisae* Mirab-balou & Chen, 2013 described by Mirab-balou and Chen (2013)

+ *Rubiothrips tongi* Mirab-balou & Chen, 2013 described by Mirab-balou and Chen (2013)

+ *Rubiothrips vitalbae* (Bagnall, 1926) added by Mirab-balou and Chen (2013)

*Rubiothrips vitis* (Priesner, 1933)

***Scirtothrips* Shull, 1909**

*Scirtothrips mangiferae* Priesner, 1932

***Scolothrips* Hinds, 1902**

*Scolothrips latipennis* Priesner, 1950

*Scolothrips longicornis* Priesner, 1926

*Scolothrips rhagebianus* Priesner, 1950

***Sitothrips* Priesner, 1931**

*Sitothrips arabicus* Priesner, 1931

***Sphaeropothrips* Priesner, 1928**

*Sphaeropothrips vittipennis* (Bagnall, 1927)

***Stenchaetothrips* Bagnall, 1926**

+ *Stenchaetothrips biformis* (Bagnall, 1913) added by Mirab-balou and Chen (2011a)

***Stenothrips* Uzel, 1895**

*Stenothrips graminum* Uzel, 1895

***Taeniothrips* Amyot & Serville, 1843**

*Taeniothrips inconsequens* (Uzel, 1895)

***Tamaricothrips* Priesner, 1964**

*Tamaricothrips tamaricis* (Bagnall, 1926)

***Tenothrips* Bhatti, 1967**

*Tenothrips discolor* (Karny, 1907)

*Tenothrips frici* (Uzel, 1895)

*Tenothrips latoides* (Pelikan, 1968)

*Tenothrips reichardti* (Priesner, 1926)

***Thermothrips* Pelikan, 1949**

+ *Thermothrips mohelensis* (Pelikan, 1949) added by Mirab-balou and Chen (2013)

***Thrips* Linnaeus, 1758**

*Thrips alavii* Mirab-balou, Tong & Chen, 2012 described by Mirab-balou et al. (2012b)

*Thrips albopilosus* Uzel, 1895

+ *Thrips alliorum* (Priesner, 1895) added by Mirab-balou et al. (2012b)

*Thrips angusticeps* Uzel, 1895

*Thrips atratus* Haliday, 1836

+ *Thrips australis* Bagnall, 1915 added by Minaei (2012b)

*Thrips dubius* Priesner, 1927

*Thrips euphorbiae* Knechtel, 1923

*Thrips flavus* Schrank, 1776

*Thrips fraudulentus* (Priesner, 1954)

*Thrips fuscipennis* Haliday, 1836

*Thrips hawaiiensis* (Morgan, 1913)

*Thrips iranicus* Yakhontov, 1951

*Thrips major* Uzel, 1895

*Thrips mareoticus* (Priesner, 1932)

*Thrips meridionalis* (Priesner, 1926)

*Thrips minutissimus* Linnaeus, 1758

*Thrips nigropilosus* Uzel, 1895

*Thrips pelikani* Schliephake, 1964

*Thrips physapus* Linnaeus, 1758

*Thrips pillichi* Priesner, 1924

*Thrips pistaciae* Yakhontov, 1951

*Thrips simplex* (Morison, 1930)

*Thrips tabaci* Lindeman, 1889

*Thrips trehernei* Priesner, 1927

*Thrips verbasci* (Priesner, 1920)

*Thrips vuilleti* (Bagnall, 1933)

*Thrips vulgatissimus* Haliday, 1836

### Suborder Tubulifera - Family Phlaeothripidae

Only a single family is recognized in this suborder, the Phlaeothripidae, with two subfamilies, Idolothripinae and Phlaeothripinae. Species of Phlaeothripidae are diverse in their biologies. Idolothripinae are all considered to feed on fungal spores (Mound and Palmer 1983). In the Phlaeothripinae, three “lineages” (*Haplothrips*, *Liothrips* and *Phlaeothrips*) have been recognized (Mound and Marullo 1996). The *Haplothrips* lineage is now well defined as the tribe Haplothripini (Mound and Minaei 2007, Minaei and Mound 2008). Members of this tribe are usually phytophagous, but some *Haplothrips* species are predators on other small arthropods, and one unusual Haplothripine species has been demonstrated to be a predator of the eggs of social wasps (Cavalleri et al. 2013). Members of the “*Phlaeothrips* lineage” are fungus feeders on fungal hyphae (Mound et al. 2013a). Species in the “*Liothrips* lineage” are leaf-feeding on the leaves of shrubs and trees, and many of these are involved in the induction of galls on leaves (Ananthakrishnan and Raman 1989). Four species in four genera of Idolothripinae, and 41 species in 15 genera of Phlaeothripinae, are recognized in Iran.

### Subfamily Idolothripinae

***Allothrips* Hood, 1908**

+ *Allothrips pillichelus bournieri* Mound, 1972 added by Minaei (2011)

***Compsothrips* Reuter, 1901**

*Compsothrips albosignatus* (Reuter, 1884)

***Megathrips*** Targioni-Tozzetti, 1881

*Megathrips flavipes* (Reuter, 1901)

***Pseudocryptothrips*** Priesner, 1919

*Pseudocryptothrips meridionalis* Priesner, 1919

### Subfamily Phlaeothripinae
Tribe Haplothripini

***Bagnalliella* Karny, 1920**

+ *Bagnalliella yuccae* (Hinds, 1902) added by Mirab-balou et al. (2012a)

***Dolicholepta* Priesner, 1932**

*Dolicholepta micrura* (Bagnall, 1914)

***Haplothrips* Amyot & Serville, 1843**

*Haplothrips aculeatus* (Fabricius, 1803)

*Haplothrips andresi* Priesner, 1931

*Haplothrips clarisetis* Priesner, 1931

*Haplothrips distinguendus* (Uzel, 1895)

*Haplothrips eragrostidis* Priesner, 1931

*Haplothrips flavicinctus* (Karny, 1910)

*Haplothrips flavitibia* Williams, 1916

*Haplothrips ganglbaueri* Schmutz, 1913

*Haplothrips globiceps* (Bagnall, 1934)

+ *Haplothrips herajius* Minaei & Aleosfoor, 2013 described by Minaei and Aleosfoor (2013)

*Haplothrips kermanensis* zur Strassen, 1975

*Haplothrips kurdjumovi* Karny, 1913

*Haplothrips leucanthemi* (Schrank, 1781)

*Haplothrips maroccanus* Priesner, 1950

*Haplothrips phyllophilus* Priesner, 1914

*Haplothrips reuteri* (Karny, 1907)

*Haplothrips subtilissimus* (Haliday, 1852)

*Haplothrips tamaricinus* Priesner, 1939

*Haplothrips tritici* (Kurdjumov, 1912)

*Haplothrips vuilleti* Priesner, 1920

***Neoheegeria* Schmutz, 1909**

*Neoheegeria dalmatica* Schmutz, 1909

*Neoheegeria gigantea* (Priesner, 1934) added by Minaei and Behmanesh (2012)

*Neoheegeria persica* Priesner, 1954

***Plicothrips* Bhatti, 1979**

*Plicothrips apicalis* (Bagnall, 1915)

***Liothrips* lineage**

***Ataliothrips* Bhatti, 1995**

*Ataliothrips reuteri* (Bagnall, 1913)

***Cephalothrips* Uzel, 1895**

*Cephalothrips coxalis* Bagnall, 1926

*Cephalothrips monilicornis* (Reuter, 1885)

***Liothrips* Uzel, 1895**

*Liothrips austriacus* (Karny, 1909)

*Liothrips jakhontovi* Kreutzberg, 1955

*Liothrips pragensis* Uzel, 1895

*Liothrips setinodis* (Reuter, 1880)

### *Phlaeothrips* lineage

***Aleurodothrips* Franklin, 1909**

+ *Aleurodothrips fasciapennis* Franklin, 1909 added by Mirab-balou and Chen (2012b)

***Hindsiothrips* Stannard, 1958**

+ *Hindsiothrips sisakhti* Minaei, 2013 described by Minaei (2013b)

***Hoplandrothrips* Hood, 1912**

*Hoplandrothrips bidens* (Bagnall, 1910)

*Hoplandrothrips hungaricus* Priesner, 1961

***Hoplothrips* Amyot & Serville, 1843**

+ An unknown species added by Jalali Sandi et al. (2011)

***Idiothrips* Faure, 1933**

+ *Idiothrips bellus* Faure, 1933 *Idiothrips ficus* Bhatti, 1967 is synonymized with *Idiothrips bellus* by Minaei (2013b)

***Phlaeothrips* Haliday, 1836**

*Phlaeothrips coriaceus* Haliday, 1836

***Stictothrips* Hood, 1924**

*Stictothrips faurei* Hood, 1924

### Unconfirmed Thysanoptera species
Aeolothripidae

***Aeolothrips***

Cheraghian and Barimani Varandi (2000) reported *Aeolothrips insularis* Priesnerfrom Iran based on a specimen identified by zur Strassen as “near *Aeolothrips insularis*” (e-mail from zur Strassen to Bhatti, see Bhatti et al. 2009a). Therefore, the record of *Aeolothrips insularis* in Iran is doubtful (see also Bhatti et al. 2009a). Moreover, the records of two other species in the genus, *Aeolothrips balati* Pelikan and *Aeolothrips citricinctus* Bagnall from Iran are also not confirmed (see Minaei 2013a).

### Phlaeothripidae

***Haplothrips***

The report of *Haplothrips minutus* (Uzel) from Iran is based on specimens identified by zur Strassen with a query (?). Similarly, the reports of three other species of *Haplothrips*, (*Haplothrips caespitis* Priesner, *Haplothrips longipes* Bagnall, and *Haplothrips rabinovitchi* Priesner) from Iran have also not been confirmed (Minaei and Mound 2008).

### Species removed from the Iranian Thysanoptera list
Thripidae

***Caliothrips striatopterus* (Kobus, 1892):** this species was recorded by Manzari (2004) in an informal newsletter as a cursory report, and so is excluded from the Iranian list (see also Bhatti et al. 2009a, Minaei and Aleosfoor 2013).

***Chaetanaphothrips* Haliday, 1836**

The only mention of this genus in an Iranian context appeared in a text book (Esmaili 1983) with these thrips noted as potential pests in the north of Iran, but no species was recorded. The species in the genus are widespread in tropical and subtropical countries, and also in greenhouses in temperate areas including Europe (zur Strassen 2003); it is possible the genus may be found in Iran as well. However, at present there is no evidence to indicate the occurrence of any species of the genus in Iran.

### Frankliniella

***Frankliniella cephalica* (Crawford DL, 1910):** the species appeared in the Iranian literature as a potential pest in the north of Iran but with no recorded details of occurrence (Esmaili 1983). *Frankliniella cephalica* has been recorded between Bermuda and Trinidad, and in Mexico and Colombia as well as Japan and Taiwan (Hoddle et al. 2013).

***Frankliniella sulphurea* Schmutz, 1913:** the species is considered as a good species by Bhatti et al. (2009a), but is usually considered a synonym of *Frankliniella schultzei*. The body colour in *Frankliniella schultzei* is variable and the species has 17 synonyms from various tropical countries around the world (Cavalleri and Mound 2012).

***Frankliniella tritici* (Fitch, 1855):** the species appeared in the Iranian literature as an external plant quarantine element (but not recorded) (Salavatian 1996). *Frankliniella tritici* is widespread in North America (Hoddle et al. 2013).

***Scirtothrips citri* (Moulton, 1909)**: the Californian citrus thrips was mentioned in the text book by Esmaili (1983) in which the author described damage to the flowers, leaves and fruits of citrus plants. However, no evidence was provided concerning the presence of this species in Iran. In addition to California, the species has been found in Arizona and Mexico (Hoddle et al. 2013).

***Scolothrips sexmaculatus* (Pergande, 1890):** the species was first reported from Iran by Shishehbor (1991) based on specimens that were not authentically determined. The name was used subsequently by a few other Iranian authors. The species identified has been recorded with certainly only from North America (including California) (Hoddle et al. 2013). According to Mound (2011b), Old World records probably all refer to *Scolothrips rhagebianus*, which has been recorded in Iran (see also Bhatti et al. 2009a).

***Thrips coloratus* Schmutz, 1913**: the species was recorded by Manzari (2004) in an informal newsletter as a cursory report and although potentially it might occur in Iran (zur Strassen 2003), it is excluded here.

### Phlaeothripdae

***Haplothrips***

***Haplothrips bagnalli* (Trybom, 1910)**

**nr. *Haplothrips bagrolis* Bhatti, 1973**

***Haplothrips cerealis* Priesner**

The first two species listed above have already been excluded from the Iranian list by Minaei and Aleosfoor (2013), whilst the third is a misidentification of *Haplothrips tritici* (Kurdjumov) (Minaei and Mound 2008). There is no evidence of the presence of *Haplothrips cerealis* in Iran (Minaei and Mound 2010b).

***Haplothrips rasouliani* Mirab-balou & Chen:** this name recently appeared in a paper (Mirab-balou et al. 2012c) but it is not available under the terms of the International Commission on Zoological Nomenclature International Commission on Zoological Nomenclature according to article 8.1, and so it is excluded here.

## Discussion

Knowledge of the natural biological systems of Iran is variable. Despite excellent floristic studies, such as Flora Iranica that now provides an identification system to more than 10,000 plant species (Rechinger 1989), comprehensive studies on the insect fauna of this country are lacking. Iran, in particular, is a bridge between the faunas of the European and Oriental Realms, and this produces considerable difficulties in studying any single group. In addition, the number of species recorded from any given area in this country almost totally depends on where particular specialists have lived or spent their careers. Consequently, field sampling of the thrips fauna of Iran has been uneven across the various Provinces, and so the results do not necessarily represent the biological diversity of any given area. Although there are a few thrips (especially those that are well known as crop pests) that are found almost all over Iran, several Provinces have yet to be surveyed for their thrips fauna. Even in those Provinces that have been apparently well-surveyed, there are still thrips species remaining to be discovered. For instance, Fars Province has been surveyed for at least 14 years continuously yet there are still several examples of recently collected material from the Province in the Collection of Department of Plant Protection, Shiraz University, that represent unrecorded, or even undescribed species. Despite this, faunistic knowledge of these tiny insects in Iran is better than in neighbouring countries, presumably due to political unrest in most of the neighbouring countries.

Although the fauna of Iran shares many species with the European Mediterranean region, other areas have a considerable effect on the Iranian fauna. For example, among the 125 species from the family Thripidae recorded here, 91 are also present in the European Mediterranean area (zur Strassen 2003). Of the remaining species, 11 have been described from Iran and most of the other 23 species are from the Oriental.
